# Cobalt complexes modulate plasmid conjugation in *Escherichia coli* and *Klebsiella pneumoniae*

**DOI:** 10.1038/s41598-024-58895-x

**Published:** 2024-04-06

**Authors:** Ilyas Alav, Parisa Pordelkhaki, Pedro Ernesto de Resende, Hannah Partington, Simon Gibbons, Rianne M. Lord, Michelle M. C. Buckner

**Affiliations:** 1 Institute of Microbiology and Infection, College of Medical and Dental Sciences, University of Birmingham, Birmingham, B15 2TT UK; 2 School of Pharmacy, Faculty of Science, University of East Anglia, Norwich Research Park, Norwich, NR4 7TJ UK; 3 Natural & Medical Sciences Research Center, University of Nizwa, Birkat Al Mauz, P.O. Box 33, Nizwa, 616 Oman; 4 School of Chemistry, Faculty of Science, University of East Anglia, Norwich Research Park, Norwich, NR4 7TJ UK

**Keywords:** Plasmid conjugation, Extended-spectrum beta-lactamase (ESBL), Carbapenemase, Anti-plasmid complexes, Antimicrobial resistance (AMR), Microbiology, Antimicrobials, Bacteria

## Abstract

Antimicrobial resistance genes (ARG), such as extended-spectrum β-lactamase (ESBL) and carbapenemase genes, are commonly carried on plasmids. Plasmids can transmit between bacteria, disseminate globally, and cause clinically important resistance. Therefore, targeting plasmids could reduce ARG prevalence, and restore the efficacy of existing antibiotics. Cobalt complexes possess diverse biological activities, including antimicrobial and anticancer properties. However, their effect on plasmid conjugation has not been explored yet. Here, we assessed the effect of four previously characterised bis(*N*-picolinamido)cobalt(II) complexes lacking antibacterial activity on plasmid conjugation in *Escherichia coli* and *Klebsiella pneumoniae.* Antimicrobial susceptibility testing of these cobalt complexes confirmed the lack of antibacterial activity in *E. coli* and *K. pneumoniae*. Liquid broth and solid agar conjugation assays were used to screen the activity of the complexes on four archetypical plasmids in *E. coli* J53. The cobalt complexes significantly reduced the conjugation of RP4, R6K, and R388 plasmids, but not pKM101, on solid agar in *E. coli* J53. Owing to their promising activity, the impact of cobalt complexes was tested on the conjugation of fluorescently tagged extended-spectrum β-lactamase encoding pCT*gfp* plasmid in *E. coli* and carbapenemase encoding pKpQIL*gfp* plasmid in *K. pneumoniae*, using flow cytometry. The complexes significantly reduced the conjugation of pKpQIL*gfp* in *K. pneumoniae* but had no impact on pCT*gfp* conjugation in *E. coli*. The cobalt complexes did not have plasmid-curing activity, suggesting that they target conjugation rather than plasmid stability. To our knowledge, this is the first study to report reduced conjugation of clinically relevant plasmids with cobalt complexes. These cobalt complexes are not cytotoxic towards mammalian cells and are not antibacterial, therefore they could be optimised and employed as inhibitors of plasmid conjugation.

## Introduction

Antimicrobial resistance (AMR) is a global public health crisis that jeopardises our ability to treat infectious diseases^1,2^. The threat of AMR has been further compounded by the dissemination of antimicrobial resistance genes (ARGs) by mobile genetic elements such as plasmids, which can carry multiple genes encoding proteins that confer resistance to a wide range of clinically relevant antibiotics^3,4^. Worryingly, opportunistic pathogens like multidrug-resistant (MDR) *Enterobacteriaceae*, such as *Escherichia coli* and *Klebsiella pneumoniae*, often harbour plasmids that carry ARGs coding for extended-spectrum β-lactamases (ESBLs e.g., CTX-M) and carbapenemases (e.g., KPC), which confer resistance to β-lactam and carbapenem antibiotics, respectively^5–8^. Hence, infections caused by MDR *Enterobacteriaceae* have become increasingly difficult to treat due to dwindling treatment options^9^. Consequently, patients with carbapenem-resistant *Enterobacteriaceae* infections face a significantly greater risk of death compared to patients with carbapenem susceptible *Enterobacteriaceae* infections^10^. In 2017, the World Health Organisation (WHO) recognised the threat posed by carbapenem-resistant and ESBL-producing *Enterobacteriaceae*. It designated this pathogen group as a critical priority for which novel drugs are needed^11^. Therefore, there is an urgent need to develop new strategies to treat these infections, including making carbapenem-resistant and ESBL-producing *Enterobacteriaceae* susceptible to existing drugs and preventing the spread of AMR between bacteria.

ARGs are commonly found in conjugative AMR plasmids that can be readily shared between bacteria that occupy the same environmental niche^12–14^. The transmission of AMR plasmids via conjugation between different species of *Enterobacteriaceae* has been well-documented in both clinical and environmental settings^15–17^. Once *Enterobacteriaceae* acquire ESBL- or carbapenemase-producing AMR plasmids, they can become MDR and extremely challenging to eradicate when they cause infections^18^. Several different factors contribute to the prevalence and spread of AMR plasmids, including high conjugation rates, increased plasmid copy number, reduced fitness cost of plasmid carriage due to compensatory mutations, and successful plasmid and clonal group interplay^19–24^. Owing to the potential to transfer multiple ARGs simultaneously, their high mobility and persistence, and the significant impact they have on treatment options, AMR plasmids are a serious threat to both animal and human health.

A potential strategy to overcome the threat of carbapenem-resistant and ESBL-producing *Enterobacteriaceae* is by reducing the prevalence of AMR plasmids^25^. This could restore the efficacy of existing well-tolerated drugs and reduce the necessity of using more toxic alternatives. Compounds that target plasmids can work by removing the plasmid from a population by reducing plasmid stability (plasmid curing) and/or interfering with the conjugation process to prevent the transfer of a plasmid to a new host^25–27^. Previous studies have identified compounds with plasmid curing/conjugation inhibiting activity, ranging from phytochemicals to clinically approved drugs^28–31^. Except for clinically approved drugs, the majority of the previously described compounds such as biocides, DNA intercalating agents, and detergents are either toxic or have not been tested in clinically relevant AMR plasmids of *Enterobacteriaceae*^25^.

Cobalt (Co) is a trace element in the body and is essential for many biological processes, and excess amounts or deficiency of the metal can induce undesired effects^32^. Cobalt complexes have found usage as anticancer, antiviral, and antimicrobial agents^33,34^. In particular, Co(II) and Co(III) complexes have been reported to have high activities against *Staphylococcus aureus* and *E. coli*^35^, and fungal strains^36^. Previously, tris(*N*-picolinamido)cobalt(III) complexes were reported to have antibacterial activity against *Pseudomonas* and *E. coli*^35^. However, Ghandhi et al.^37^ reported the ESKAPE screening (CO-ADD; Community for Open Antimicrobial Drug Discovery, The University of Queensland, Australia) of a range of bis(*N*-picolinamido)cobalt(II) complexes that had antifungal activity but minimal antibacterial activity and importantly, no cytotoxicity against mammalian cell lines^37^. These results suggest the oxidation state of the cobalt complexes could induce differences in their bacterial mode of action. Although the antimicrobial effects of cobalt complexes have been explored, their impact on plasmid conjugation has not been studied to date. Here, we evaluated the activity of four previously characterised cobalt complexes^37^ lacking antibacterial activity for their ability to reduce the conjugation of various plasmid types in *E. coli* J53. The most promising compounds were then tested for their impact on the conjugation of ESBL- or carbapenemase-producing plasmids tagged with *gfp* by flow cytometric analysis in clinical *E. coli* and *K. pneumoniae* isolates, respectively.

## Materials and methods

### Strains and plasmids

All bacterial strains and plasmids used in this study are listed in Tables 1 and 2, respectively. Unless stated otherwise, all strains were grown in Luria–Bertani (LB) broth/broth with agar (Merck, Germany) at 37 °C with aeration (liquid cultures).

**Table 1:** List of bacterial strains used in this study.

Species	Features	Source of strain
*Escherichia coli*	NCTC 10418/ATCC 10536 susceptible reference strain	NCTC
*Staphylococcus aureus*	NCTC 12981/ATCC 25923 susceptible reference strain, weak β-lactamase producer	NCTC
*Escherichia coli*	*Escherichia coli* J53 with the conjugative pKM101 plasmid, which confers resistance to β-lactams	DSMZ GmbH
*Escherichia coli*	*Escherichia coli* J53 with the conjugative R388 plasmid, which confers resistance to trimethoprim and sulphonamides	DSMZ GmbH
*Escherichia coli*	*Escherichia coli* J53 with the conjugative R6K plasmid, which confers resistance to β-lactams and streptomycin	DSMZ GmbH
*Escherichia coli*	*Escherichia coli* J53 carrying the conjugative RP4 plasmid, which confers resistance to β-lactams, kanamycin, and tetracycline	DSMZ GmbH
*Escherichia coli*	*Escherichia coli* J53 with the hygromycin resistance gene *hph* inserted into the *att*Tn*7* site (Hyg^R^)	This study
*Klebsiella pneumoniae*	*Klebsiella pneumoniae* Ecl8 with *mCherry-aph* inserted into chromosomal *putPA* intergenic region. Resistant to kanamycin and expresses mCherry constitutively	^30^
*Klebsiella pneumoniae*	*Klebsiella pneumoniae* Ecl8 carrying pKpQIL with *gfp-aph* inserted into the *bla*_KPC_ gene. Resistant to kanamycin and expresses GFP constitutively	^30^
*Escherichia coli*	*Escherichia coli* ST131 EC958c with *mCherry-aph* inserted into chromosomal *putPA* intergenic region. Resistant to kanamycin and expresses mCherry constitutively	^30^
*Escherichia coli*	*Escherichia coli* ST131 EC958c carrying pCT with *gfp-aph* inserted into the *bla*_CTX-M-14_ gene. Resistant to kanamycin and expresses GFP constitutively	^30^

Hyg^R^, hygromycin resistant. DSMZ GmbH strains were obtained from the German Collection of Microorganisms and Cell Cultures, and NCTC strains were obtained from the National Collection of Type Cultures.

**Table 2:** List of plasmids used in this study.

Plasmid	Description	References
pKM101	Conjugative IncN plasmid derived from the clinically isolated R46 plasmid. It contains the *bla*_OXA-2_ resistance gene that confers resistance to β-lactams	^65,66^
R388	Conjugative IncW plasmid isolated from clinical samples of *E. coli* and *Klebsiella* spp. It contains *dfrb2* and *sul1* genes that confer resistance to trimethoprim and sulphonamide, respectively	^67,68^
R6K	Conjugative IncX2 plasmid with MOB_P3_ type relaxase. It contains *strAB* and *bla*_TEM-1_ resistance genes that confer resistance to streptomycin and β-lactams, respectively	^69,70^
RP4	Conjugative IncP plasmid with a broad host range isolated from drug-resistant *P. aeruginosa* clinical isolates. It contains *bla*_TEM-1,_ *aph(3’)-Ib*, and *tetA* genes that confer resistance to β-lactams, kanamycin, and tetracycline, respectively	^71,72^
pSIM18	Plasmid used as a template to amplify the hygromycin resistance gene	^73^
pSLTS	Plasmid encoding arabinose inducible lambda red recombination system to facilitate homologous recombination	^41^
pCT*gfp*	Conjugative IncK plasmid pCT initially isolated from cattle. It has *gfp-aph* inserted into the *bla*_CTX-M-14_ gene for constitute GFP expression and selection with kanamycin	^5,30^
pKpQIL*gfp*	Conjugative IncFII_K2_ plasmid pKpQIL responsible for carbapenem resistance in outbreaks of multidrug resistant *K. pneumoniae*. It has *gfp-aph* inserted into the *bla*_KPC_ gene for constitute GFP expression and selection with kanamycin	^8,30^

### Determination of antibacterial susceptibility

The broth microdilution method was used to determine the minimum inhibitory concentrations (MICs) of ampicillin and the cobalt complexes Co4, Co5, Co6, and Co8 ranging from 1 to 512 µg/mL according to Clinical and Laboratory Standards Institute guidance^38^. Ampicillin was included as a control antibiotic for the antimicrobial susceptibility testing as the MIC values for the *E. coli* NCTC 10418 and *S. aureus* NCTC 12981 (Table 1) quality control strains are known^38^. The MIC values were recorded as the lowest concentration at which no bacterial growth was detected. All MICs were carried out using three biological replicates.

### Growth kinetic assays

The impact of the cobalt complexes on bacterial growth was determined as previously described^39^. Briefly, overnight cultures (~ 10^9^ CFU/mL) of the strains used for the conjugation assays were diluted to a starting inoculum of 10^6^ CFU/mL in a 96-well flat bottom plate (Corning, USA). Where appropriate, the test strains were diluted in LB broth supplemented with the cobalt complexes or DMSO vehicle control to a final concentration of 100 µg/mL. Growth was monitored at OD_600_ at 30-min intervals for 12 h using the FLUOstar OMEGA plate reader (BMG Labtech, Germany). Three independent experiments were carried out, each consisting of three biological replicates.

### Construction of the hygromycin-resistant *Escherichia coli* J53 recipient strain

To obtain a hygromycin-resistant *E. coli* J53 strain to be used as a recipient for conjugation assays, the *hph* gene encoding hygromycin B phosphotransferase was inserted into the phenotypically neutral *att*Tn*7* site^40^ using the arabinose inducible recombineering plasmid pSLTS as described previously^41^. Firstly, the *hph* gene was amplified from the pSIM18 plasmid using primers that have flanking 40 bp homology to the *att*Tn*7* site in *E. coli* (Supplementary Table S1). The arabinose inducible recombineering plasmid pSLTS was electroporated into *E. coli* J53 with subsequent electroporation of the PCR-amplified hygromycin resistance cassette. Successful recombinants were selected on LB agar supplemented with 150 µg/mL hygromycin. PCR and Sanger sequencing (Eurofins Genomics, UK) using primers that bind upstream and downstream of the recombination site (Supplementary Table S1), were used to verify the successful insertion of the *hph* gene at the desired genomic locus. Antimicrobial susceptibility testing was also used to verify the hygromycin-resistant phenotype (MIC > 512 µg/mL).

### Liquid broth conjugation assay

The donor *E. coli* J53 strain with R388, pKM101, RP4 or R6K was paired with the hygromycin-resistant recipient strain *E. coli* J53 *att*Tn*7*::*hph*. The liquid broth conjugation assays were performed as previously described with minor modifications^42^. Donor and recipient cultures were grown overnight, sub-cultures were prepared in 5 mL LB broth (1% inoculum) and grown to an OD_600_ of ~ 0.5. A 1 mL culture volume was pelleted, and media were replaced with LB broth to normalise the OD_600_ to 0.5. Equal volumes of donor and recipient strains were mixed to give a donor-to-recipient ratio of 1:1. Cultures were diluted 1:5 in LB broth containing a final concentration of 100 µg/mL of cobalt complexes or 100 µg/mL DMSO as vehicle control. These were incubated statically at 37 °C for 4 h. Cells were serially diluted in sterile phosphate-buffered saline (PBS) (10^–1^ to 10^–6^) and plated on selective media and incubated at 37 °C overnight. Transconjugant colonies carrying RP4, R6K or pKM101 were selected on LB agar supplemented with 150 µg/mL hygromycin B (PhytoTech Labs, USA) and 100 µg/mL carbenicillin (Merck, Germany). Transconjugant colonies carrying R388 were selected on LB agar supplemented with 150 µg/mL hygromycin B and 10 µg/mL trimethoprim (Merck, Germany). Conjugation frequencies (CF) were calculated using the following formula:

$${\text{Conjugation}}\;{\text{frequency}} = \frac{{{\text{mean}}\;{\text{number}}\;{\text{of}}\;{\text{transconjugants}}}}{{{\text{mean}}\;{\text{number}}\;{\text{of}}\;{\text{recipients}} \times {\text{donor}}/{\text{recipient}}\;{\text{ratio}}}}$$

Three independent experiments were carried out, each one consisting of four biological replicates.

### Solid agar conjugation assay

The donor *E. coli* J53 strains with R388, pKM101, RP4 or R6K were paired with the hygromycin-resistant recipient strain *E. coli* J53 *att*Tn*7*::*hph*. The solid agar conjugation assay was performed as previously described with minor modifications^43^. Briefly, a 1 mL volume of overnight cultures of donor and recipient cells was pelleted, washed with LB broth, and the OD_600_ was adjusted to 0.5. Equal volumes of donor and recipient cells were mixed to give a donor-to-recipient ratio of 1:1. Then, 5 µL of this mixture, which contained bacteria at an OD_600_ of 0.5, was placed on top of 96-well round bottom plates (Corning, USA) containing 150 µL LB agar supplemented with 100 µg/mL of cobalt complexes or 100 µg/mL DMSO as vehicle control. Conjugation was carried out for 4 h at 37 °C without agitation. Bacteria were resuspended in 150 µL sterile PBS and diluted cells (10^–1^ to 10^–6^) were plated on selective media as described above and incubated at 37 °C overnight. Conjugation frequencies were calculated the same way as for the liquid conjugation assay. Three independent experiments were carried out, each one consisting of four biological replicates.

### Measurement of plasmid conjugation by flow cytometry

The conjugation of pCT*gfp* in *E. coli* ST131 EC958c and pKpQIL*gfp* in *K. pneumoniae* Ecl8 was measured by flow cytometry as previously described^30^. In our experience, bacteria grown on solid agar surfaces are less suited to flow cytometry than liquid cultures as the bacteria tend to form clumps and doublets. Therefore, the conjugation of pCT*gfp* in *E. coli* ST131 EC958c and pKpQIL*gfp* in *K. pneumoniae* Ecl8 was determined in liquid broth. Briefly, 1 mL overnight cultures of the donor (*E. coli* with pCT*gfp* or *K. pneumoniae* with pKpQIL*gfp*) and the recipient (*E. coli* or *K. pneumoniae* with chromosomal mCherry) strains were pelleted, washed in sterile PBS, and diluted to an OD_600_ of 0.5. Equal volumes of donor and recipient strains were mixed to give a donor-to-recipient ratio of 1:1. A 20 µL volume of the donor-recipient mix was inoculated into 180 µL of LB broth supplemented with a final concentration of 100 µg/mL of cobalt complexes or 100 µg/mL DMSO as vehicle control in a 96-well round bottom plate (Corning, USA). The plate was incubated at 37 °C with gentle agitation (∼100 rpm) for 4 h. Following incubation, 20 µL was removed and serially diluted 1:1000 in filter-sterilised Dulbecco’s PBS (Merck, Germany). Samples were analysed on the Attune NxT acoustic focusing flow cytometer with Autosampler (Thermo Scientific, USA). GFP emission was collected using the BL1-H channel and the mCherry emission was collected using the YL2-H channel. Plasmid conjugation was measured by quantifying the number of green fluorescent protein (GFP)-positive bacteria (donor), mCherry-positive bacteria (recipient), and GFP-positive/mCherry-positive bacteria (transconjugants). Gating strategies were exactly as previously described^30^. Plasmid conjugation was calculated as the number of dual fluorescent bacterial events divided by the total bacterial events relative to the DMSO control. Three independent experiments were carried out, each one consisting of four biological replicates.

### Assessment of plasmid-curing activity

Overnight cultures of *E. coli* J53 carrying RP4, R6K, R388, or pKM101, *E. coli* ST131 EC958c carrying pCT*gfp*, and *K. pneumoniae* Ecl8 carrying pKpQIL*gfp* were grown. Sub-cultures were prepared in 5 mL LB broth (5% inoculum) and grown to an OD_600_ of ~ 0.6. A 1 mL volume of culture was pelleted, and media were replaced with LB broth to normalise the OD_600_ to 0.5. A 10 µL volume of culture was inoculated into 190 µL of LB broth supplemented with a final concentration of 100 µg/mL cobalt complexes or 100 µg/mL DMSO as vehicle control in a 96-well round bottom plate (Corning, USA). The plate was then incubated at 37 °C for 24 h without agitation. Following 24 h incubation, each well was serially diluted to 10^–6^ in sterile PBS. A 10 µL volume of the 10^–6^ diluted culture was then used to passage the cells in 190 µL LB broth supplemented with a final concentration of 100 µg/mL cobalt complexes or 100 µg/mL DMSO as vehicle control in a 96-well round bottom plate for a further 24 h incubation. This dilution factor was used to impose a bottleneck on the population during passage experiments. Cells were serially diluted in sterile PBS (10^–1^ to 10^–6^) and the first (24 h) and second passage (48 h) post-inoculation were plated onto both selective media and non-selective media and incubated at 37 °C overnight. The plasmids RP4, R6K, and R388 were selected on 100 µg/mL carbenicillin, R388 on 10 µg/mL trimethoprim, and pCT*gfp* and pKpQIL*gfp* on 50 µg/mL kanamycin. The percentage of plasmid persistence was calculated as

$${\text{Plasmid}}\;{\text{persistence}} = \frac{{{\text{mean}}\;{\text{number}}\;{\text{of}}\;{\text{cells}}\;{\text{on}}\;{\text{selective}}\;{\text{media}}}}{{{\text{mean}}\;{\text{number}}\;{\text{of}}\;{\text{cells}}\;{\text{on}}\;{\text{non - selective}}\;{\text{media}}}} \times 100$$

Three independent experiments were carried out, each one consisting of four biological replicates.

### Statistical analysis

Unpaired *t*-tests were used for statistical analysis with GraphPad Prism version 10 for MacOS, San Diego, California USA, http://www.graphpad.com. Only P-values less than or equal to 0.05 were considered statistically significant.

## Results

### Cobalt complexes are not antibacterial

Firstly, the susceptibility of the quality control and the strains used for the plasmid conjugation assays (Table 1) to the cobalt complexes Co4, Co5, Co6, and Co8 (Supplementary Fig. S1) was determined to detect any antibacterial activity and to identify a suitable concentration to evaluate their effect on plasmid conjugation. In agreement with the previous study^37^, none of the tested cobalt complexes exhibited antibacterial activity against the strains tested up to a concentration of 512 µg/mL (Supplementary Table S2). Preliminary work showed that 100 µg/mL was the lowest concentration which showed activity in plasmid conjugation assays. Therefore, the effect of the cobalt complexes on plasmid conjugation was tested at 100 µg/mL. Before the plasmid conjugation assays, and to ensure 100 µg/mL had no impact on growth, bacterial growth kinetics in the presence of 100 µg/mL cobalt complexes were compared to 100 µg/mL DMSO vehicle control. These experiments showed that none of the cobalt complexes had any significant adverse effects on bacterial growth over 12 h (Supplementary Fig. S2).

### Cobalt complexes affected plasmid conjugation differently in liquid broth and solid agar mating

**Figure 1:**
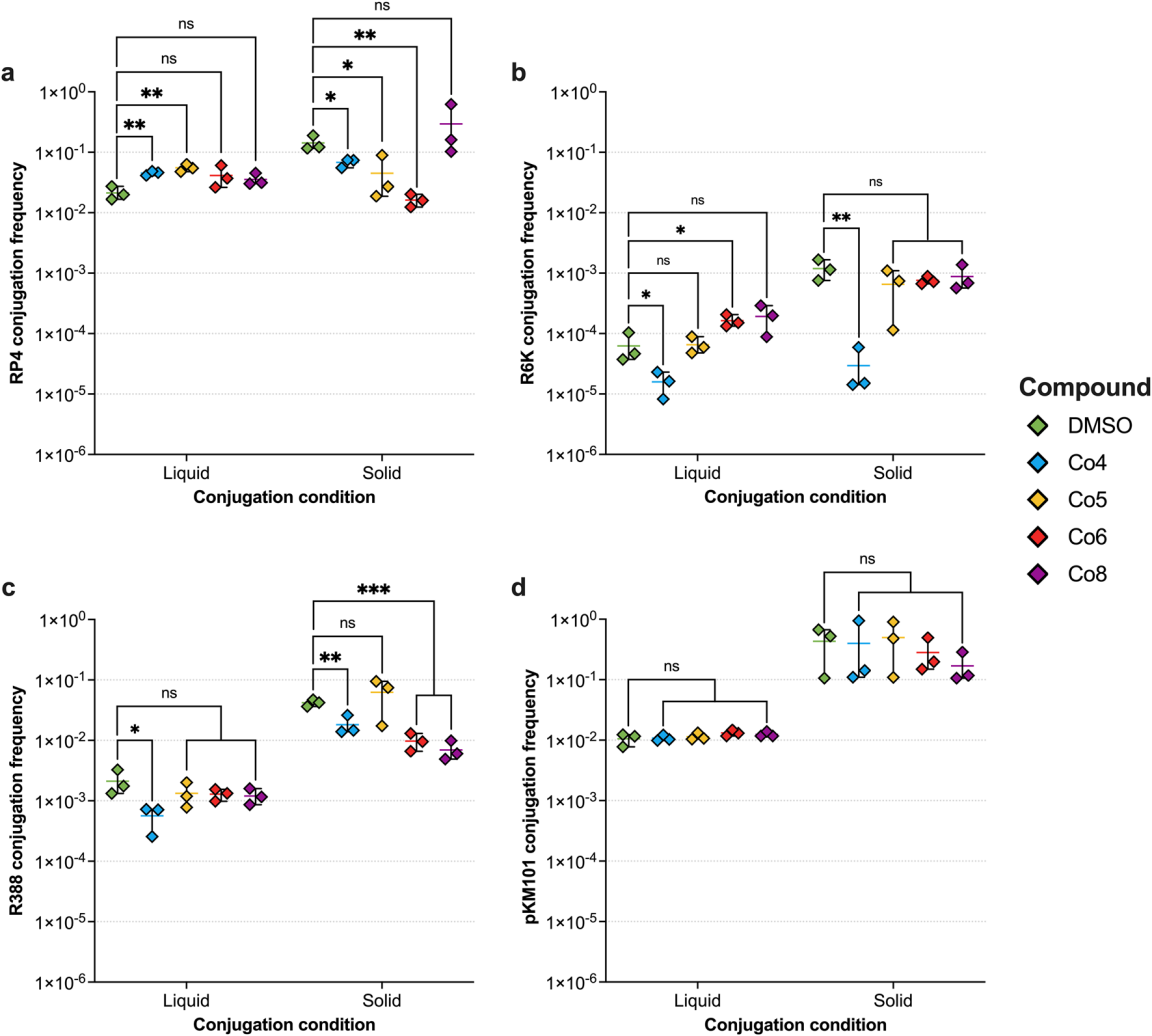
The effect of cobalt complexes on the conjugation frequencies of plasmids with different incompatibility groups in liquid LB broth and on LB agar. Conjugation frequencies of (**a**) the IncP plasmid RP4, (**b**) the IncX2 plasmid R6K, (**c**) the IncW R388, and (**d**) the IncN plasmid pKM101, from *E. coli* J53 to hygromycin resistant *E. coli* J53 *att*Tn*7*::*hph* in the presence of 100 µg/mL DMSO vehicle control or 100 µg/mL cobalt compound after four-hour incubation. Data shown are the mean ± standard deviation of three independent experiments, each carried out with four biological replicates. Cobalt complexes that significantly affected conjugation frequency compared to DMSO control are indicated with * (*p* ≤ 0.05), ** (*p* ≤ 0.01) or *** (*p* ≤ 0.001). ns, not significant.

The cobalt complexes were first screened using a panel of *E. coli* J53 strains carrying different plasmid types (Table 1). Plasmid conjugation frequencies (CF) are also known to differ in liquid broth and on solid surfaces^44^. Therefore, the effect of cobalt complexes on plasmid conjugation was tested using both liquid broth and agar mating experiments. In agreement with previous studies^44,45^, the IncP plasmid RP4, the IncX2 plasmid R6K, the IncW plasmid R388 and the IncN plasmid pKM101 displayed higher CFs on solid agar compared to liquid broth mating (Fig. 1 and Table 3).

**Table 3:** Conjugation frequency data for RP4, R6K, R388 and pKM101 plasmids in liquid broth and solid agar mating in the presence of 100 µg/mL cobalt compounds or DMSO vehicle control.

Compound	Conjugation frequency							
	RP4		R6K		R388		pKM101	
	Solid	Liquid	Solid	Liquid	Solid	Liquid	Solid	Liquid
DMSO	4.26 × 10^–3^ ± 1.10 × 10^–3^	4.26 × 10^–3^ ± 1.10 × 10^–3^	1.19 × 10^–3^ ± 4.59 × 10^–4^	6.26 × 10^–5^ ± 3.62 × 10^–5^	4.19 × 10^–2^ ± 5.3 × 10^–3^	2.11 × 10^–3^ ± 1.02 × 10^–3^	4.33 × 10^–1^ ± 2.93 × 10^–1^	1.06 × 10^–2^ ± 2.44 × 10^–3^
Co4	6.79 × 10^–2^ ± 1.08 × 10^–2^	9.06 × 10^–3^ ± 7.39 × 10^–4^	2.95 × 10^–5^ ± 2.56 × 10^–5^	1.58 × 10^–5^ ± 7.40 × 10^–6^	1.82 × 10^–2^ ± 6.85 × 10^–3^	5.63 × 10^–4^ ± 2.66 × 10^–4^	3.99 × 10^–1^ ± 4.73 × 10^–1^	1.08 × 10^–2^ ± 1.24 × 10^–3^
Co5	4.50 × 10^–2^ ± 3.85 × 10^–2^	1.11 × 10^–2^ ± 1.57 × 10^–3^	6.52 × 10^–4^ ± 4.99 × 10^–4^	6.53 × 10^–5^ ± 2.12 × 10^–5^	6.23 × 10^–2^ ± 4.02 × 10^–2^	1.33 × 10^–3^ ± 6.26 × 10^–4^	4.97 × 10^–1^ ± 3.96 × 10^–1^	1.14 × 10^–2^ ± 1.54 × 10^–3^
Co6	1.62 × 10^–2^ ± 3,91 × 10^–3^	8.42 × 10^–3^ ± 3.51 × 10^–3^	7.59 × 10^–4^ ± 1.16 × 10^–4^	1.63 × 10^–4^ ± 3.80 × 10^–5^	9.71 × 10^–3^ ± 3.20 × 10^–3^	1.29 × 10^–3^ ± 2.86 × 10^–4^	2.81 × 10^–1^ ± 1.88 × 10^–1^	1.32 × 10^–2^ ± 1.56 × 10^–3^
Co8	2.95 × 10^–1^ ± 2.84 × 10^–1^	7.11 × 10^–3^ ± 1.66 × 10^–3^	8.78 × 10^–4^ ± 4.39 × 10^–4^	1.92 × 10^–4^ ± 1.01 × 10^–4^	6.49 × 10^–3^ ± 2.61 × 10^–3^	1.20 × 10^–3^ ± 3.65 × 10^–4^	1.70 × 10^–1^ ± 1.01 × 10^–1^	1.24 × 10^–2^ ± 1.27 × 10^–3^

Conjugation frequencies shown are the mean of three independent experiments ± standard deviation, each carried out with four biological replicates. Conjugation frequency was calculated as the number of transconjugants/(number of recipients × donor-to-recipient ratio.

Interestingly, the cobalt complexes affected plasmid CFs differently depending on the conjugation condition. Co4 and Co5 significantly increased the CF of RP4 in liquid broth mating, whilst in solid agar mating they both significantly reduced the CF of RP4 (Fig. 1a) from 1.42 × 10^–1^ in DMSO control to 6.79 × 10^–2^ in Co4 (*p* = 0.0186) and 4.49 × 10^–2^ in Co5 (*p* = 0.0197) (Table 3). Whilst Co6 had no impact on the CF of RP4 in liquid broth (Fig. 1a), it significantly reduced CF in solid agar mating from 1.42 × 10^–1^ in DMSO control to 1.61 × 10^–2^ in Co6 (*p* = 0.0029) (Table 3). Co8 did not affect RP4 CF in liquid broth and solid agar mating (Fig. 1a). For R6K and R388, Co4 significantly reduced their CF in both conditions, but the reduction in CF was more pronounced in solid agar mating (R6K, *p* = 0.0062 and R388, *p* = 0.0048) (Fig. 1b and c). Co6 had no significant impact on R6K CF in solid agar mating (Fig. 1b) but significantly increased CF from 6.26 × 10^–5^ in DMSO control to 1.63 × 10^–4^ in liquid broth (*p* = 0.0146) (Table 3). Additionally, Co6 and Co8 had no significant impact on the CF of R388 in liquid broth (Fig. 1c), but significantly reduced its CF in solid agar mating from 4.19 × 10^–2^ in DMSO control to 9.71 × 10^–3^ (*p* = 0.0004) and 6.49 × 10^–3^ (*p* = 0.0003) in Co6 and Co8, respectively (Table 3). None of the cobalt complexes had a significant impact on the CF of pKM101 in both liquid broth and solid agar mating (Fig. 1d), indicating that IncN plasmids are possibly not targeted by cobalt complexes.

### Impact of cobalt complexes on the conjugation of plasmids carrying extended-spectrum β-lactamase and carbapenemase genes

**Figure 2:**
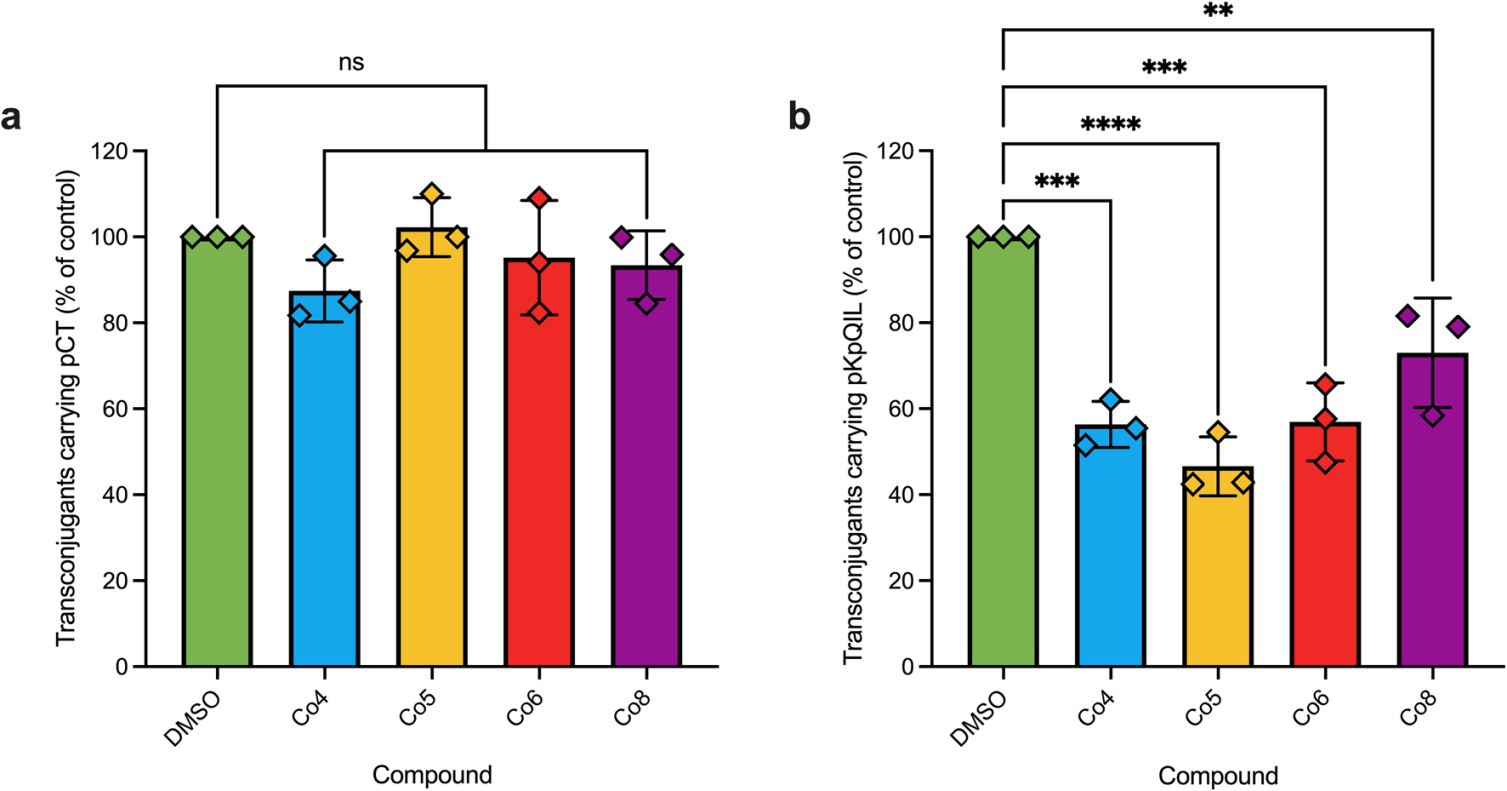
The impact of cobalt complexes on the conjugation of plasmids carrying extended spectrum β-lactamase and carbapenemase genes measured by flow cytometry. The percentage of transconjugant (**a**) *Escherichia coli* ST131 EC958c carrying pCT*gfp* and (**b**) *Klebsiella pneumoniae* Ecl8 carrying pKpQIL*gfp*, following incubation with 100 µg/mL cobalt compound compared to 100 µg/mL DMSO vehicle control after 4 h incubation. The data shown are the mean ± standard deviation from three independent experiments, each carried out with four biological replicates. Cobalt complexes that significantly reduced the percentage of transconjugant bacteria compared to DMSO control are indicated with ** (*p* ≤ 0.01) or *** (*p* ≤ 0.001). ns, not significant.

After confirming anti-plasmid activity in the J53 *E. coli* isolates carrying three of the four plasmids (RP4, R6K, and R388), we wanted to assess their activity in clinical bacterial isolates with plasmids conferring resistance to ESBLs and carbapenems. To test the impact of the four cobalt complexes on the conjugation of clinically relevant strains/plasmids using a previously developed liquid-conjugation assay that uses flow cytometry to quantify transconjugants^30^. The recipient *E. coli* and *K. pneumoniae* strains expressed a chromosomal *mCherry* gene whilst the donor *E. coli* strain carrying the IncK type plasmid pCT or the *K. pneumoniae* strain carrying IncFII type plasmid pKpQIL were tagged with constitutively active *gfp*. In this setup, transconjugant bacteria were measured based on their dual fluorescence of GFP and mCherry proteins. None of the four cobalt complexes had a significant effect on the percentage of transconjugant *E. coli* carrying pCT*gfp* compared to DMSO control (Fig. 2a), suggesting this IncK plasmid was not the target of the tested cobalt complexes in this study. On the other hand, all four cobalt complexes significantly reduced the percentage of transconjugant *K. pneumoniae* carrying pKpQIL*gfp* compared to DMSO control (Fig. 2b).

### Cobalt complexes do not have plasmid-curing activity

**Figure 3:**
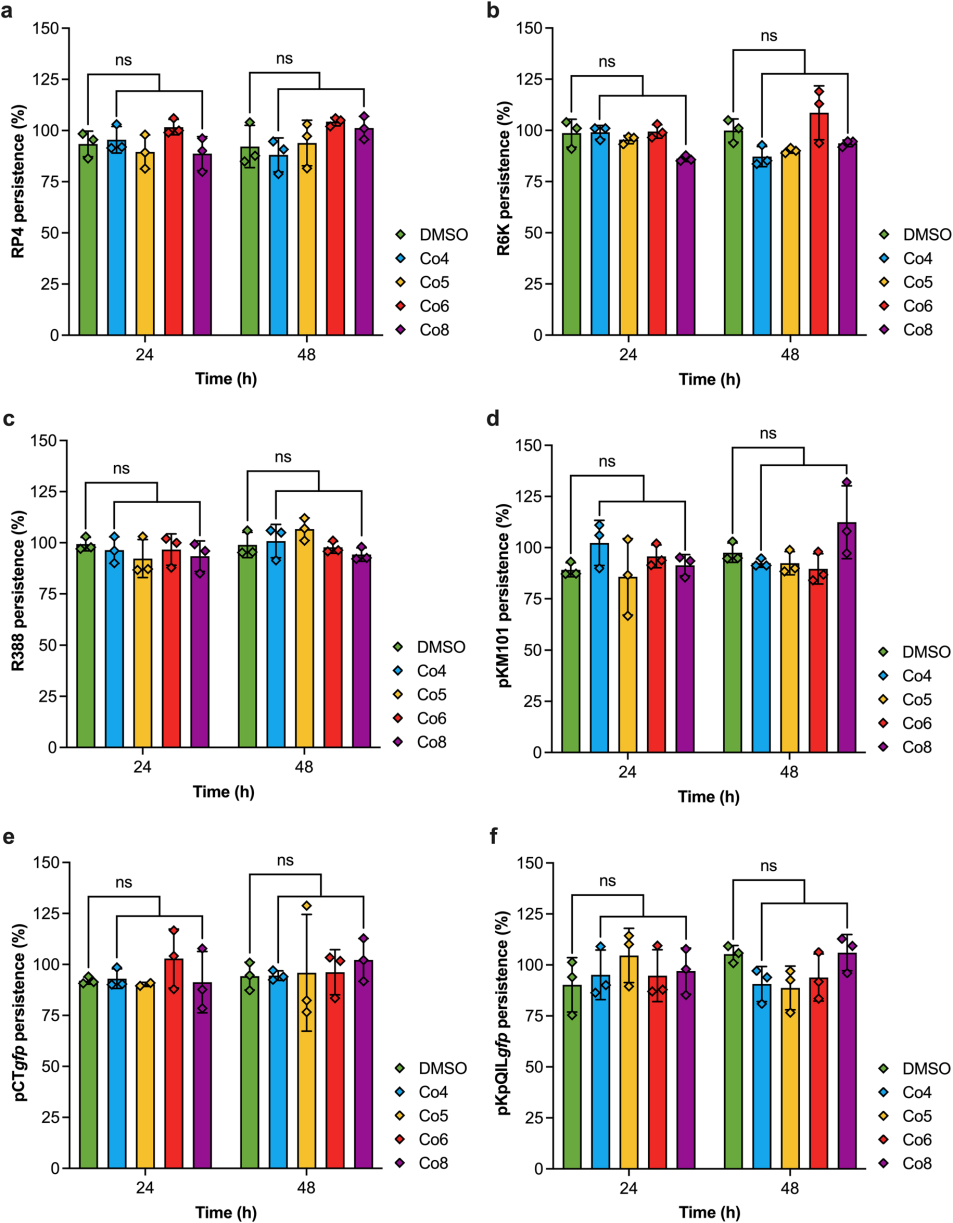
The effect of cobalt complexes on plasmid persistence. The persistence of (**a**) the IncP plasmid RP4, (**b**) the IncX2 plasmid R6K, (**c**) the IncW R388, (**d**) the IncN plasmid pKM101, (**e**) the IncK plasmid pCT with tagged with a *gfp* gene, and (**f**) the IncFII plasmid pKpQIL tagged with a *gfp* gene, in the presence of 100 µg/mL of cobalt complexes after 24 and 48 h compared to 100 µg/mL DMSO control. The data shown are the mean ± standard deviation from three independent experiments, each conducted with four biological replicates. ns, not significant.

To determine whether the cobalt complexes affected plasmid stability and maintenance, the impact of the cobalt complexes as plasmid curing agents was measured over 48 h. This time frame was chosen to reflect the change in transconjugant production observed over the conjugation assays (4- and 24-h), as a compound which cures a donor strain of the plasmid would generate fewer transconjugants in a conjugation assay. The DMSO (100 µg/mL) control did not affect plasmid persistence after 24 and 48 h (Fig. 3). The cobalt complexes did not display plasmid-curing activity against any of the six plasmids compared to the DMSO control after 24 and 48 h (Fig. 3). This suggested that the cobalt complexes affected the conjugation process rather than plasmid persistence in the donor bacteria.

## Discussion

The rise in AMR combined with the dwindling pipeline of new antibiotics in development warrants novel strategies to combat the AMR crisis^2^. Plasmids play a key role in the global dissemination of AMR genes in MDR Gram-negative bacteria^3^. Targeting plasmids is a novel strategy to combat AMR by reducing the prevalence of AMR genes and sensitising bacteria to existing antibiotics^25^. In addition, such complexes could be used in a One-Health setting by removing or reducing AMR genes in animals and/or the environment^3^.

Metal ion complexes represent an increasing trend in the development of antimicrobial agents^46^. Cobalt complexes have essential biochemical functions and have been reported to possess antibacterial, antifungal, and antiviral properties^37,47^. However, the impact of cobalt complexes on plasmid conjugation has never been explored. In this study, four previously characterised bis(*N*-picolinamido)cobalt(II) complexes (Co4, Co5, Co6, and Co8) were assessed for their ability to reduce conjugation of different plasmids and to see whether they exhibited plasmid-curing activity. The results showed that the cobalt complexes did not have a plasmid-curing effect on the tested plasmid types after 48 h. Previous studies that have investigated plasmid-curing agents reported significant plasmid elimination after 18 – 48 hr^48–50^. Hence, the cobalt complexes were likely to be affecting the conjugation process rather than ridding bacterial cells of their residing plasmid.

Plasmids persist in cells through different mechanisms including toxin-antitoxin, restriction-modification, and entry-exclusion systems^51^. These ensure plasmids are stably maintained in bacterial cells during cell division (e.g. through toxin-mediated killing of plasmid-free daughter cells) and conjugative plasmid transfer^51^. Therefore, the lack of activity of the cobalt complexes on plasmid stability could be attributed to the diverse persistence mechanisms that act to maintain plasmids during cell division. It is plausible that such mechanisms were responsible for the high level of plasmid stability seen in our assays, but effective curing compounds must be able to overcome these mechanisms.

The conjugation frequencies of RP4, R6K, R388, and pKM101 plasmids were higher on solid agar compared to liquid broth mating. This could be partly due to the differences in the dilution rates used during the experimental setup between liquid broth and solid agar mating, as well as the changes in the lifestyle of bacteria (growing in a biofilm on solid agar versus planktonic culture in liquid broth), which is known to impact conjugation rates^42^. The cobalt complexes were most effective at reducing plasmid conjugation on solid agar, rather than in liquid mating assays. Complexes Co4 and Co5 significantly increased the conjugation frequency of RP4 and Co6 increased the conjugation frequency of R6K in liquid broth (Fig. 1). On the other hand, complexes Co4, Co5, and Co6 significantly reduced the conjugation frequency of RP4 on solid agar (Fig. 1). Similarly, Co4, Co6, and Co8 significantly reduced R388 conjugation on solid agar but only Co4 reduced R388 conjugation in liquid broth as well (Fig. 1). The IncP plasmid RP4 and the IncW plasmid R388 have been previously shown to have constitutive rigid pilus synthesis that plays an important role in conjugation on solid surfaces^44^. Cobalt complexes that reduced conjugation specifically on solid agar may target plasmid-specific pilus formation/assembly to impede donor-recipient contact and transfer of single-stranded plasmid DNA^52^. For the IncX2 plasmid R6K, only Co4 significantly reduced its conjugation frequency in both liquid broth and on solid agar. Indeed, Co4 reduced solid agar conjugations of 3/4 plasmids, with only pKM101 (which was not affected by any compound in solid or liquid assays) showing no impact. Hence, Co4 may target a shared component of the type 4 secretion systems (T4SSs) that mediates the conjugative transfer of plasmid DNA from the donor to the recipient cell.

Co4 possesses diisothiocyanato ligands that could explain its effect on plasmid conjugation. Isothiocyanates have been shown to affect cell membrane potential and bacterial redox systems^53^. The morphogenesis of T4SSs requires both the proton motive force and ATP energy^54^. Therefore, the diisothiocyanato ligands of Co4 may potentially interfere with the assembly and function of T4SSs by disrupting the membrane potential. Cobalt has multiple effects on bacterial physiology and metabolism^55^. Some plasmids encode proteins with metal-binding domains that could potentially be targeted by cobalt. The single-stranded DNA-binding protein ArdC, encoded by the R388 plasmid, is important for conjugation and depends on manganese binding for its activity^56^. However, cobalt(II) ions also bind to the active site of ArdC and inhibit its activity^56^. Conjugative plasmids encode T4SS primases like TraC of RP4 that contain metallopeptidase domains and relaxases like TraI of RP4 that contain magnesium-binding sites. Cobalt(II) can occupy the same binding site as magnesium or manganese to form different coordination bonds and alter the properties of an active centre^57^. Hence, cobalt complexes may target metal-binding T4SS proteins and interfere with their function.

All four cobalt complexes significantly reduced the conjugation of the IncFII plasmid pKpQIL in *K. pneumoniae* as measured by flow cytometry (Fig. 2b). Therefore, it is plausible that they have a common target that is necessary for successful conjugative plasmid transfer, such as the type 4 secretion system^58^, or a common effect on *K. pneumoniae.* The cobalt complexes did not impact the growth kinetics of *K. pneumoniae* strains. The cobalt complexes had no significant effect on the pKM101 conjugation frequency in both solid agar and liquid broth (Fig. 1d). They also did not affect the conjugation of the IncK plasmid pCT in *E. coli* as measured by flow cytometry (Fig. 2a). These results suggested that the cobalt complexes did not target these IncK and IncN plasmids. This is possibly due to diverse elements in the conjugation apparatus between different plasmid incompatibility groups^43,59–61^.

None of the cobalt complexes exhibited antibacterial activity (Supplementary Table S2), which corroborates with the previously reported data^37^. Moreover, the previous study demonstrated that these cobalt complexes have no cytotoxicity towards mammalian cells^37^. To date, this is the first description of cobalt complexes that reduced plasmid conjugation. The efficacy of the cobalt complexes on plasmid conjugation on solid agar as opposed to in liquid broth suggests that they could be candidates for inhibitors of plasmid conjugation in solid or semi-solid environments where bacteria reside, such as biofilms on hospital surfaces, plumbing, and indwelling surfaces^62–64^. Further work involving structural modification and mechanism of action studies on bis(*N*-picolinamido)cobalt(II) complexes could potentially lead to the development of broad-range conjugation inhibitors.

### Supplementary Information

Supplementary Information.

## Data Availability

The authors confirm that the data supporting the findings of this study are available within the article and its supplementary materials.
