# Supplementary material for: Cobalt complexes modulate plasmid conjugation in Escherichia coli and Klebsiella pneumoniae
Source: Sci Rep. 2024 Apr 6;14:8103. doi: 10.1038/s41598-024-58895-x (PMC10998897; doi:10.1038/s41598-024-58895-x)
Supplement: Supplementary file 1 — Supplementary Information. [file 41598_2024_58895_MOESM1_ESM.pdf]

Supplementary materials

Cobalt complexes modulate plasmid conjugation in *Escherichia coli* and *Klebsiella pneumoniae*

Ilyas Alav[1], Parisa Pordelkhaki[1], Pedro Ernesto de Resende[2], Hannah Partington[1], Simon Gibbons[3], Rianne Lord[4], Michelle M.C. Buckner[1#]

[1] Institute of Microbiology and Infection, College of Medical and Dental Sciences, University of Birmingham, B15 2TT, Birmingham, UK

[2] School of Pharmacy, Faculty of Science, University of East Anglia, Norwich Research Park, NR4 7TJ, Norwich, UK

[3] Natural & Medical Sciences Research Center, University of Nizwa, P.O. Box 33, Birkat Al Mauz, Nizwa 616, Oman

[4] School of Chemistry, Faculty of Science, University of East Anglia, Norwich Research Park, NR4 7TJ, Norwich, UK

[#] Corresponding author.

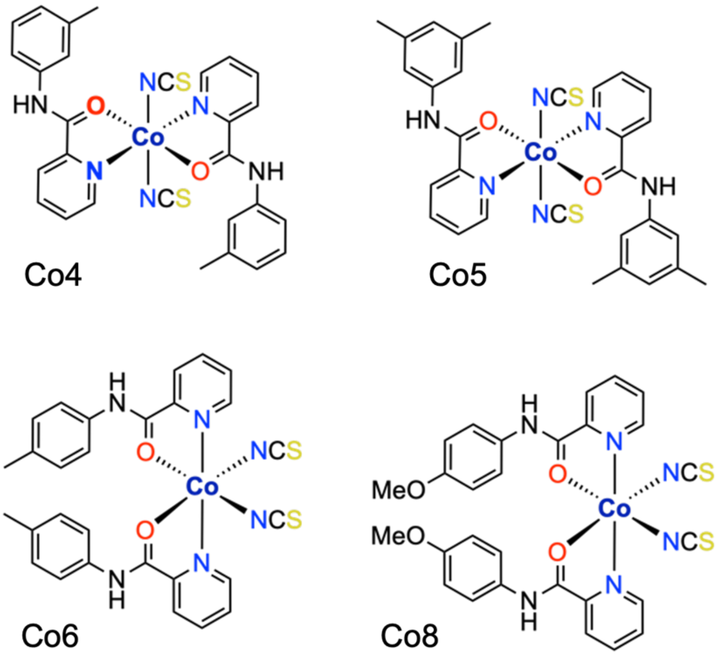

Figure S1. The structures of bis(*N*-picolinamido)cobalt(II) complexes Co4, Co5, Co6, and Co8. All complexes have been drawn with the geometries obtained from single-crystal X-ray diffraction data[1].

Table S1. Primers used in this study.

Primer ID	Description	Sequence (5'-3')
P77	Forward primer for generation of the hygromycin resistance cassette from pSIM18 with flanking 40 bp homology (underlined) to the target locus of the *Tn*7-transposon downstream of the *glmS* gene	TCGGTTACGGTTGAGTAATAAATGGATGCCCTGCGTAAGCGCATTCAAATATGTATCCGCT
P78	Reverse primer for generation of the hygromycin resistance cassette from pSIM18 with flanking 40 bp homology (underlined) to the target locus of the *Tn*7-transposon downstream of the *glmS* gene	AGGATGTTTGATTAAAAACATAACAGGAAGAAAAATGCCCCTATTCCTTTGCCCTCGGACG
P87	Forward primer that binds upstream of the inserted hygromycin resistance gene in the *E. coli* J53 chromosome	GCACCGAACAACGAATTGCT
P88	Reverse primer that binds downstream of the inserted hygromycin resistance gene in the *E. coli* J53 chromosome	TGTTTTACGCCACCGGAAGA
P153	Forward primer that binds to *oriT* on the RP4 plasmid	CAGGATTCCCGTTGAGCACC
P154	Reverse primer that binds to *oriT* on the RP4 plasmid	TCTTTGGCATCGTCTCTCGC
P155	Forward primer that binds to *rep1* on the pKM101 plasmid	GTTCTTCTGTTGGGATTCCG
P156	Reverse primer that binds to *rep1* on the pKM101 plasmid	GCGAAGATGATGATGAGATGG
P157	Forward primer that binds to γ *ori* on the R6K plasmid	CTAAGGGCTTCTCAGTGCGT
P158	Reverse primer that binds to γ *ori* on the R6K plasmid	CGCTATATTACCCCAAACCCG
P159	Forward primer that binds to *repA* on the R388 plasmid	CAGGAACACGCGATAGGTCA
P160	Reverse primer that binds to *repA* on the R388 plasmid	TCACCTTGTCGATCATGGGC

Table S2. Susceptibility of the test strains to the bis(*N*-picolinamido)cobalt(II) complexes.

	**MIC (µg/mL)**				
**Strain**	AMP	Co4	Co5	Co6	Co8
S. aureus NCTC 12981	1	>512	>512	>512	>512
E. coli NCTC 10418	4	>512	>512	>512	>512
E. coli J53 *att*Tn*7*::*hph*	4	>512	>512	>512	>512
E. coli J53 with RP4	>512	>512	>512	>512	>512
E. coli J53 with R6K	>512	>512	>512	>512	>512
E. coli J53 with R388	4	>512	>512	>512	>512
E. coli J53 with pKM101	>512	>512	>512	>512	>512
K. pneumoniae Ecl8 *mCherry*	ND	>512	>512	>512	>512
K. pneumoniae Ecl8 with pKpQIL*gfp*	ND	>512	>512	>512	>512
E. coli ST131 EC958c *mCherry*	ND	>512	>512	>512	>512
E. coli ST131 EC958c with pCT*gfp*	ND	>512	>512	>512	>512

The minimum inhibitory concentration (MIC) values shown are the median of three biological replicates. AMP, ampicillin. ND indicates that data were not required and so not determined.

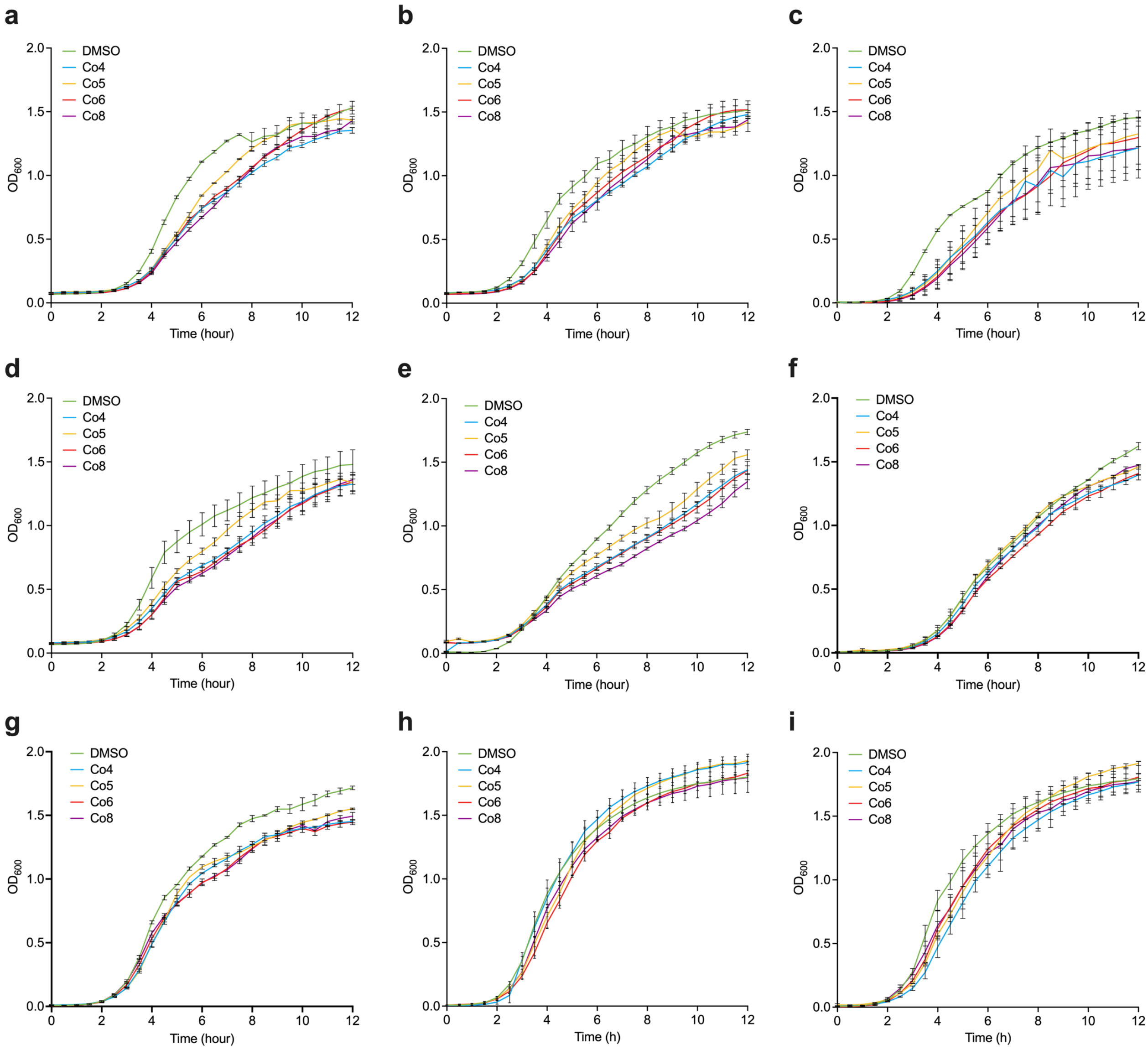

Figure S2. The effect of cobalt compounds on the growth of bacterial strains. Growth kinetics of **(a)** hygromycin-resistant *E. coli* J53 *att*Tn*7*::*hph*, **(b)** *E. coli* J53 carrying IncP plasmid RP4, **(c)** *E. coli* J53 carrying IncX2 plasmid R6K, **(d)** *E. coli* J53 carrying IncW R388, **(e)** and *E. coli* J53 carrying the IncN plasmid pKM101, **(f)** *K. pneumoniae* Ecl8 mCherry, **(g)** *K. pneumoniae* Ecl8 carrying pKpQIL*gfp*, **(h)** *E. coli* ST131 carrying pCT*gfp,* and **(i)** *E. coli* ST131 mCherry, in the presence of LB broth supplemented with 100 µg/mL of cobalt compounds or 100 µg/mL DMSO. Data shown are the mean ± standard deviation of three independent experiments, each carried out with three biological replicates.

References

1 Ghandhi, L. H. D., Bidula, S., Pask, C. M., Lord, R. M. & McGowan, P. C. Bis(*N*-picolinamido)cobalt(II) Complexes Display Antifungal Activity toward *Candida albicans* and *Aspergillus fumigatus*. *ChemMedChem* **16**, 3210-3221 (2021). https://doi.org:10.1002/cmdc.202100159
